# Acetylcholinesterase-inhibiting Alkaloids from *Zephyranthes concolor*

**DOI:** 10.3390/molecules16119520

**Published:** 2011-11-15

**Authors:** Ricardo Reyes-Chilpa, Strahil Berkov, Simón Hernández-Ortega, Christopher K. Jankowski, Sebastien Arseneau, Imma Clotet-Codina, José A. Esté, Carles Codina, Francesc Viladomat, Jaume Bastida

**Affiliations:** 1 Instituto de Química, Universidad Nacional Autónoma de México, Coyoacán, 04510, México, D.F., Mexico; E-Mail: simonho@unam.mx (S.H.-O.); 2 AgroBioInstitute, 8 Dragan Tzankov Blvd., 1164 Sofia, Bulgaria; E-Mail: berkov_str@yahoo.com (S.B.); 3 Département de Chimie et Biochimie, Université de Moncton, Moncton, NB E1A 3E9, Canada; E-Mails: krzysztof.jankowski@umoncton.ca (C.K.J.); sebastien.arsenau@umocnton.ca (S.A.); 4 Laboratori de Retrovirologia, Fundació Irsi Caixa, Hospital Universitari Germans Trias i Pujol. 08916-Badalona, Barcelona, Spain; E-Mail: jaeste@irsicaixa.es (J.A.E.); 5 Departament de Productes Naturals, Biología Vegetal i Edafologia, Facultat de Farmàcia, Universitat de Barcelona. Av. Diagonal 643, E-08028 Barcelona, Spain; E-Mails: carlescodina@ub.edu (C.C.); viladomat@ub.edu (F.V.)

**Keywords:** alkaloids, chlidanthine, *Zephyranthes concolor*, amaryllidaceae

## Abstract

The bulbs and aerial parts of *Zephyranthes concolor* (Lindl.) Benth. & Hook. f. (Amaryllidaceae), an endemic species to Mexico, were found to contain the alkaloids chlidanthine, galanthamine, galanthamine N-oxide, lycorine, galwesine, and epinorgalanthamine. Since currently only partial and low resolution ^1^H-NMR data for chlidanthine acetate are available, and none for chlidanthine, its 1D and 2D high resolution ^1^H- and ^13^C-NMR spectra were recorded. Unambiguous assignations were achieved with HMBC, and HSQC experiments, and its structure was corroborated by X-ray diffraction. Minimum energy conformation for structures of chlidanthine, and its positional isomer galanthamine, were calculated by molecular modelling. Galanthamine is a well known acetylcholinesterase inhibitor; therefore, the isolated alkaloids were tested for this activity. Chlidanthine and galanthamine N-oxide inhibited electric eel acetylcholinesterase (2.4 and 2.6 × 10^−5^ M, respectively), indicating they are about five times less potent than galanthamine, while galwesine was inactive at 10^−3^ M. Inhibitory activity of HIV-1 replication, and cytotoxicity of the isolated alkaloids were evaluated in human MT-4 cells; however, the alkaloids showed poor activity as compared with standard anti-HIV drugs, but most of them were not cytotoxic.

## 1. Introduction

The genus *Zephyranthes* Herb. (Amaryllidaceae, tribe Hippeastreae) is composed of approximately 60 species distributed in the American continent. A number of species are cultivated due to their gorgeous flowers, and are known by plant breeders as “rain lilies”, owing this name to their tendency to flower after a rain period [1]. *Zephyranthes concolor* (Lindl.) Benth. & Hook. f . (synonym: *Habranthus concolor* Lindl.) is a species endemic of Mexico [2], whose chemical composition is currently unknown. To our best knowledge, ten *Zephyranthes* species have been chemically studied (*Z. andersoniana* [3], *Z. candida* (Lindl.) Herb. [4,5], *Z. carinata* Herb. [6], *Z citrina* Baker [7], *Z. flava* (Herb.) Baker [8], *Z. grandiflora* Lindl. [9], *Z. robusta* (Herb. ex Sweet) Baker [10], Z. *rosea* Lindl. [11], *Z. sulphurea* hort. [10], and *Z. tubispatha* (L’Hér.) Herb. [12]) and most of the isolated alkaloids belong to the lycorine type.

Amaryllidaceae alkaloids are restricted to this family. They are best known due to galanthamine (**2**), a selective and reversible inhibitor of acetylcholinesterase that increases the levels of acetylcholine in the brain, and used therefore in the treatment of Alzheimer’s disease. Galanthamine hydrobromide is currently used as an approved drug (Razydine^®^, Reminyl^®^) for the cognitive symptoms of dementia [13,14]. Compounds structurally related to galanthamine may have similar pharmacological properties; for instance, a recent survey of 23 amaryllidaceous alkaloids, showed that seven of them bearing galanthamine and lycorine skeletons exhibited remarkable acetylcholinesterase inhibitory activity *in vitro* [15].

It has been reported that several Amaryllidaceae alkaloids, like lycorine, haemanthamine, trisphaeridine, and homolycorine, can inhibit replication of human immunodeficiency virus type 1 (HIV-1) *in vitro* [16]. Lycorine, homolycorine [16], as well as littoraline, isolated from *Hymenocallis littoralis* (Jacq.) Salisb. inhibited HIV-1 reverse transcriptase [17]. The methanol extract from the roots of *Crinum asiaticum* L., known to contain lycorine type alkaloids [18], has been reported to have potent anti-HIV-1 activity, and low cytoxicity; it inhibited viral reverse transcriptase, but not protease [19]. Finally, other alkaloids from this class, such as *trans*-dihydronarciclasine and pancratistatin obtained from *Z. candida*, and *Z. grandiflora*, respectively, have shown antineoplastic properties [5,9].

**Figure 1:**
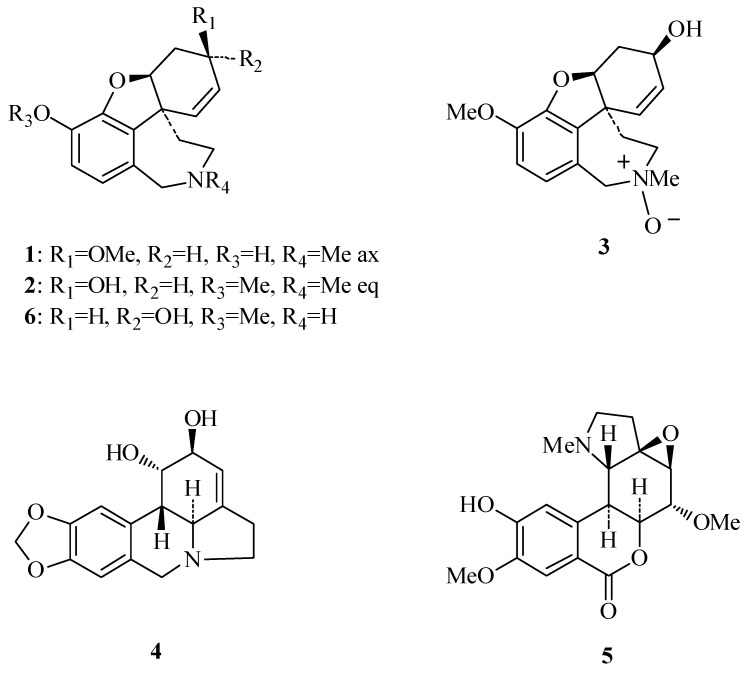
Alkaloids isolated from *Zephyranthes concolor*.

We now report on the isolation and identification of six Amaryllidaceae alkaloids (Figure 1) from the bulbs, and aerial parts of *Zephyranthes concolor*. Their identification was determined by means of spectroscopic and spectrometric methods. Since currently only partial and low resolution ^1^H-NMR data are available for chlidanthine acetate, and none for chlidanthine (**1**), its 1D and 2D high resolution ^1^H- and ^13^C-NMR spectra are reported here. Unambiguous assignations were achieved with HMBC and HSQC experiments, and its structure corroborated by X-ray diffraction. Minimum energy conformation for structures of chlidanthine (**1**), and galanthamine (**2**), were calculated by molecular modelling. The ability of the isolated compounds to inhibit electric eel acetylcholinesterase, and HIV-1 replication *in vitro* was also tested.

## 2. Results and Discussion

### 2.1. Identification of Compounds

The bulbs of *Zephyranthes concolor* contain the alkaloids chlidanthine (**1**), galanthamine (**2**), galanthamine N-oxide (**3**), lycorine (**4**), and galwesine (**5**). The first three alkaloids were also obtained from the aerial parts of the plant, along with epinorgalanthamine (**6**) (Figure 1). The isolated alkaloids belong to the galanthamine (compounds **1**, **2**, **3** and **6**), and lycorine (compounds **4** and **5**) types. Other *Zephyranthes* species studied have shown mainly the presence of lycorine type alkaloids [3,4,5,6,7,8,9,10,11]. Whereas galanthamine (**2**) is synthesized by several Amaryllidaceae species, its positional isomer chlidanthine (**1**) has only been described from three other species, in addition to the present finding in *Z. concolor*. Chlidanthine (**1**) was first isolated more than fifty years ago from *Chlidanthus fragrans* Herb. [20], and afterwards from *Haemanthus multilflorus* Martyn [21], and *Hippeastrum aulicum* var. *robustum* (A. Dietr. ex Walp.) Voss [22]. Its structure was deduced mainly by chemical methods, and currently only partial ^1^H data (60 MHz) of its acetate derivative are available [23,24,25]. Chlidanthine’s biological properties have not been studied until now.

### 2.2. Structure of Chlidanthine *(**1**)*

The ^1^H-NMR spectrum of chlidanthine (**1**) (Table 1) resembled that of galanthamine (**2**), and showed the expected structural features. The most evident was the characteristic singlet at 3.37 ppm indicative of a methoxy substituent on the allylic C-3. In general, the proton resonances of 1 appeared slightly shifted to higher magnetic fields as compared with **2**. The most important differences in chemical shifts for compound **1** were those for protons H-1, H-2β, H-3, H-4a, and H-7 with values of −0.11, −0.11, −0.34, +0.13 and −0.17 ppm, respectively. The ^13^C-NMR spectrum of compound **1** (Table 1) showed signals for seventeen carbon atoms. The DEPT experiment indicated that two of them were methyls, four methylenes, six methines and five quaternary carbon atoms. The HMBC experiment showed couplings of H-3 with C-4 (^2^*J*), C-1 and OCH_3_ (both ^3^*J*), as well as the coupling of methoxy protons with C-3 (^3^*J*) (Table 1), thus corroborating the position of OMe on C-3 of chlidanthine (**1**). The NOESY experiment indicated that H-4a interacts with H-4, H-6β, and H-12β, meanwhile NMe does it with H-6α, H-11α, and H-12α (Table 1). Comparing the ^13^C-NMR spectra of **1** and **2**, most of the carbon atoms show similar chemical shifts; but in the case of **1**, resonance signals for C-3, C-8 and C-10 appeared 8.0 and 4.3 ppm downfield, and 3.9 ppm upfield, respectively. The downfield shift observed for C-3 in **1** can be attributed to deshielding effect of the OCH_3_ substituent. The mass spectrum showed the molecular ion at *m/z* = 287, which was also the base peak, and congruent for C_17_H_21_NO.

**Figure 2:**
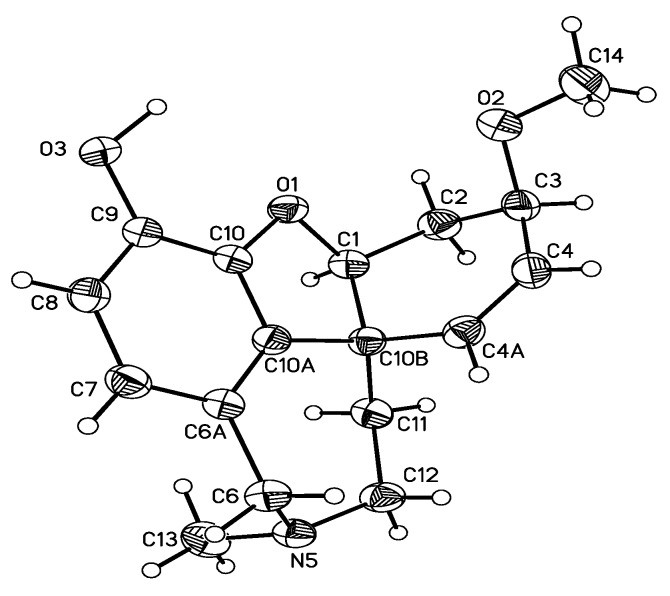
Crystal structure of chlidanthine (**1**).

The crystal structure of chlidanthine (**1**) was determined by X-ray diffraction (Figure 2), and showed that the tetrahydroazepine ring is in a chair conformation, the dihydrofuran ring in an envelope conformation, and the cyclohexene ring adapted a conformation somehow between an envelope and half-chair. The *N*-methyl group is axial, while in the case of galanthamine (**2**) it has been reported as equatorial [13,26]. In the case of galanthamine bromide, the *N*-methyl group has been described in the axial position [26]. The methoxy group of chlidanthine (**1**) is on the same side of the dihydrofuran oxygen, and both are *anti* to the *N*-methyl group.

**Figure 3:**
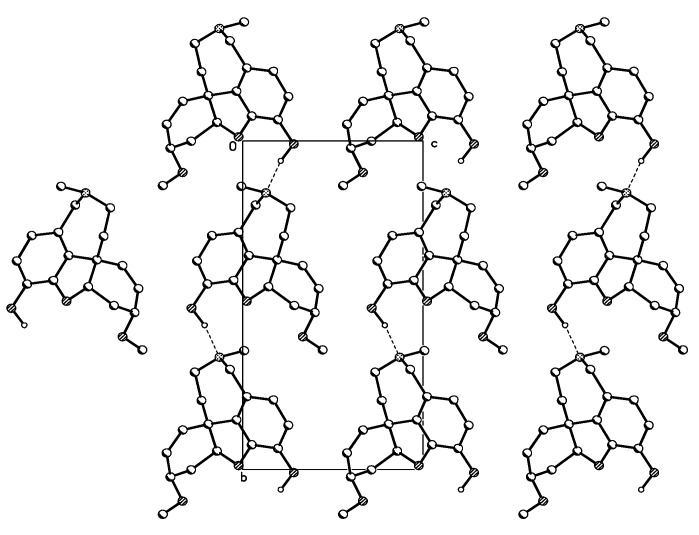
Crystal packing of chlidanthine (**1**) shows intermolecular hydrogen bonding.

The crystal packing of chlidanthine (**1**) showed intermolecular hydrogen bonding along the b axis. Therefore the hydrogen of the hydroxyl group of one molecule is bonded with the nitrogen of the tetrahydroazepine ring of a second molecule (Figure 3). In the case of galanthamine (**2**), the same pattern of intermolecular hydrogen binding (O–H^..…^N) is also known to occur [27]. In the solid state, no intramolecular hydrogen bonding was observed for chlidanthine (**1**). The IR spectrum of chlidanthine (**1**) registered in CCl_4_, a solvent in which only intramolecular hydrogen bonding can usually be observed, did not show the diagnostic band at 3574 cm^−1^ [27], thus suggesting that intramolecular hydrogen bonding could also be absent in solution.

### 2.3. Molecular Mechanics of Chlidanthine *(**1**)* and Galanthamine *(**2**)*

Molecular mechanics modelling (MM+) in gas phase was performed looking for: a) the possibility of formation of H-bond within the phenolic ether-phenol system for chlidanthine (**1**), compared to the phenolic ether-secondary alcohol one for galanthamine (**2**); b) the pyramidal inversion on the methylated nitrogen N-5 leading to two distinct orientations of the lone electron pair in space at this center. In the case of chlidanthine (**1**), the 2.7 Å distance between phenol and ether oxygen allows the consideration of an H-bond, which should be a stability factor absent for galanthamine (**2**) because of the longer distance of 4.2 Å. This observation is also in agreement with the more acidic character of the phenol group than the secondary alcohol, the hypothetical 5 or 6 member ring formed between these elements being of equal stability values. The privileged conformation of the phenolic OH group will then direct the H toward the oxygen ether atom. As far as the N-5 conformation is concerned the electronic doublet orientation found for both conformations are quite close, 1 kcal mol^−1^ of difference, but seem to privilege its α orientation (Table 2). The comparison by molecular modelling shows that **2** is slightly (2 kcal mol^−1^) more stable than **1**, and that the nitrogen lone electronic doublet is oriented in the α direction. Puzzled by these results we modeled the virtual series of alkaloids, those of four analogous *cis* junction isomers, but built with the inversion of the geometry at C-1 and C-10b (Table 2). In this virtual series, when the N-5 electronic doublet is oriented in α direction, the isomer is slightly more stable. However, the H-bond between the alcohol group and the ether cannot be formed because of the bothering β oriented H-1. All four hypothetical alkaloids (**7**, **7a**, **8** and **8a**), like **1** and **2**, have similar energies with differences of less than 1 kcal mol^–1^ within this group (Table 2). It seems then possible that the structure solvation plays a role in the conformation distributions, especially when the N-5 orientation is involved, and therefore more modelling studies, for example in solvent box, are required to definitely solve this problem.

### 2.4. Biological Activity

Chlidanthine (**1**), and galanthamine *N*-oxide (**3**) inhibited electric eel acetylcholinesterase *in vitro*, with EC_50_ = 2.41 and 2.61 × 10^−5^ M, respectively (Table 3). These alkaloids were thus 4.6 and 5.0 times less potent, respectively, than the positive control galanthamine (**2**). Galwesine (**5**) that possesses a homolycorine skeleton, was not active at 10^−3^ M (Table 3). Epinorgalanthamine’s (**6**) inhibitory properties (9.60 × 10^−6^ M) had been previously reported, while lycorine (**4)** has been found to be inactive [15]. Our results are in concordance with previous findings, since Amaryllidaceae alkaloids with galanthamine and lycorine type skeleta (with the exception of lycorine itself) have been found able to inhibit of electric eel acetylcholinesterase *in vitro*, while those of the haemanthamine, homolycorine and tazzetine classes do not [15]. Regarding the galanthamine type alkaloids, until now three alkaloids more potent than galanthamine (**2**) have been found. It was first described that the loss of the Me group at C-9, as in sanguinine, leads to about a 10-fold increase of the AChE inhibitory activity [15]. Recently, it has been reported that a similar increase in potency also occurs when the *N*-atom is substituted with an allyl group, such as in *n*-allylnorgalanthamine, and *n*-(14-methylallyl)norgalanthamine [14]. On the other hand, the loss of methyl group at the *N*-atom in epinorgalanthamine leads to a nearly 10-fold decrease of the activity. The activity is completely lost in molecules of galanthamine type with hydrogenated C4-C4a double bonds (lycoramine and epinorlycoramine) [15].

Since chlidanthine and galantamine are positional isomers, we focused on their structure-activity relationships. The existence of an intramolecular hydrogen bond between the hydroxyl on C-3 and the oxygen of the dihydrofuran ring of galanthamine (**2**), has been considered to play a major role in its acetylcholinesterase inhibitory activity [27]. In the case of chlidanthine (**1**) which lacks this feature, inhibition of acetylcholinestease was reduced. Galanthamine derivatives where the hydroxyl group was protected as a carbamate, oxidized, or suffered inversion of its configuration, also showed a decrease in acetylcholinesterase inhibitory activity [13]. Currently, studies by X-ray diffraction suggest that the relatively tight binding of galanthamine (**2**) to acetylcholinesterase of *Torpedo californica* Ayres, arises from a number of moderate to weak interactions, coupled to a low entropy cost for binding due to the rigid structure of the inhibitor. These interactions are polar, but also, noteworthy, non polar. The former involve hydrogen bonding between the hydroxyl, the oxygen atom of the dihydrofuran ring, and the *N*-methyl group of galanthamine (**2**) with different aminoacids of the enzyme, and also involve connecting water molecules [28]. The reduction in potency of chlidanthine (**1**), and galanthamine *N*-oxide (**3**) as inhibitors of acetylcholinesterase are in accordance to this hypothesis.

Results of antiviral assays indicated that none of the alkaloids here tested were able to inhibit in a significant way the replication of HIV-1 (NL4-3) in lymphoid MT-4 human cells, as compared with the potency of control anti-HIV-1 drugs, but most these alkaloids were also not toxic to non infected cells (Table 4). Lycorine (**4**) has been previously studied in this context, reporting anti-HIV-1 activity (ID_50_ = 0.4 μg/mL), and cytotoxicity (TC_50_ = 0.75 μg/mL) in human MT4 cells; therefore with a low therapeutic index (TI_50_ = 1.9) [16]. Other Amaryllidaceae alkaloids, such as homolycorine, trisphaeri-dine, and haemanthamine, have also been tested for anti-HIV-1 activity, but have shown even lower therapeutic indices than lycorine (**4**) [16]. According to a recent review, no other Amaryllidaceae alkaloids have been studied to date regarding to their possible anti-HIV-1 properties [34].

## 3. Experimental

### 3.1. General

Optical rotations were registered in EtOH solution using a Perkin Elmer polarimeter (model 343) at room temperature. IR spectra were recorded with a Bruker Tensor 27 spectrophotometer. ^1^H- and ^13^C- 1D and 2D NMR spectra were recorded on Varian spectrometers (Gemini-400 and VXR-500) by standard procedures. The chemical shifts are expressed in parts per million (δ, ppm) and tetramethylsilane (TMS) was used as internal standard. EI-MS was registered on an Agilent 5973 mass spectrometer equipped with SIS Direct Insertion Probe at an ionization voltage of 70 eV. X ray diffraction pattern was determined in a Bruker AXS Smart Apex diffractometer equipped with bidimentional area detector CCD. Silica gel 40-60 mesh, (Merck) was used for column chromatography. TLC was performed on silica gel plates (60 F254, 0.2 mm) developed with a mobile phase containing EtOAc-MeOH-25% ammonia (3:1:0.1, v/v/v), compounds on the plate were observed under UV light (254 and 365 nm) and visualized by spraying with Dragendorff´s reagent.

### 3.2. Plant Material

*Zephyranthes concolor* (Lindl.) Benth. & Hook. f. (Amaryllidaceae) was collected from the buffering area of the Ecological Reserve in the campus of Universidad Nacional Autónoma de México (UNAM) at Mexico City. Plants were found growing in abundance on soil pockets among lava fields, many of them in the flowering or fruiting stage. A voucher specimen (N. Diego 9605) was deposited in the FECME Herbarium (Facultad de Ciencias, UNAM), and identified according to published botanical keys for local flora [29]. Species names quoted along this paper are written as in the original references; therefore, they could be synonyms rather than accepted names for the taxa described.

### 3.3. Extraction and Isolation

#### 3.3.1. Bulbs

Fresh bulbs (3 kg) were cut in pieces and macerated twice during 15 days in ethanol (6 L). The extract was concentrated in a rotary evaporator and processed as described below. The dry extract was dissolved in 2% H_2_SO_4_ (700 mL), defatted with diethyl ether (5 × 500 mL), and made alkaline to pH 9–10 with 25% ammonia. Then, the alkaloids were extracted with EtOAc and EtOAc/MeOH. The extracts were evaporated under vacuum and tested for alkaloids on TLC (silica Gel 60, EtOAc/MeOH 3:1). The EtOAc extract (15 g) was Dragendorff´s positive, and was further fractionated by column chromatography (Silica Gel 60) eluting with EtOAc gradually enriched with MeOH. Fractions of 20 mL were collected and monitored by TLC obtaining four main fractions: A (fractions 14–33, EtOAc/MeOH 3:1), B (fractions 44–47, EtOAc/MeOH 1:1), C (fractions 48–102, EtOAc/MeOH 1:3) and D (fractions 103–107, MeOH). Fraction A dissolved in MeOH lead to spontaneous crystallization of lycorine (**4**, 57 mg), while mother liquors after CC (silica Gel 60, EtOAc/MeOH 3:1) yielded galwesine (**5**, 31 mg). Fraction B was subjected to CC (silica Gel 60, EtOAc/MeOH 3:1), and afforded galanthamine (**2**, 272 mg), which was further recrystallized in acetone. Chlidanthine (**1**, 50 mg) crystallized spontaneously from fraction C dissolved in MeOH, while galanthamine-*N* oxide (**3**, 83 mg) was obtained after CC (silica Gel 60, EtOAc/MeOH 1:3) of the mother liquors.

#### 3.3.2. Aerial Parts

The fresh aerial parts (0.9 kg) were cut in pieces and macerated twice during 15 days in ethanol (3 L). The extract was concentrated in a rotary evaporator. The dry extract was dissolved in 2% H_2_SO_4_, defatted with diethyl ether, and made alkaline with 25% ammonia to pH 9–10. Then, the alkaloids were extracted with EtOAc and EtOAc/MeOH. The extracts were evaporated under vacuum and tested for alkaloids on TLC (silica Gel 60, EtOAc/MeOH 3:1). Only the EtOAc was positive for alkaloids, and was further fractionated by CC (silica Gel 60). Galanthamine (**2**, 31 mg) and chlidanthine (**1**, 30 mg) were crystallized from fractions eluted with EtOAc/EtOH 1:1, and 1:3, respectively. Epinorgalanthamine (**6**, 34 mg), and galanthamine *N*-oxide (**3**, 25 mg) were obtained from fractions eluted with MeOH subjected to preparative TLC (silica Gel 60, EtOAc/MeOH 1:3).

### 3.4. Identification of Compounds

*Chlidanthine* (**1**). Colourless needles (recrystallized from acetone). [α]^D^_20_ = −129° (EtOH, c. 3.0 mg/mL). IR (CCl_4_, film) ν_max_ cm^−1^ 2923, 2853, 1604, 1460, 1254, 1088. EI-MS *m/z* (%): 287(100) M^+^ (C_17_H_21_NO), 272(9) M^+^−Me, 256(65) M^+^−OMe, 244(10), 230(6), 225(14), 212(29), 202(45) (C_17_H_12_NO_2_), 115(42), 77(39). For ^1^H- and ^13^C-NMR spectral data see Table 1.

*Galanthamine* (**2**). The ^1^H- and ^13^C-NMR spectra data were as reported by Carrol *et al*. [27].

*Galanthamine N-oxide* (**3**). The ^1^H- and ^13^C-NMR spectra data were as reported by Kobayashi *et al*. [30].

*Galwesine* (**5**). ^1^H- and ^13^C-NMR spectral data were as reported by Latvala *et al.* [31]. EI-MS *m/z* (%) 361 (12.9), 207 (1.8), 206 (2.5), 178 (7.4), 163 (1.3), 155 (90.2), 140 (100), 126 (2.7), 112 (17.4), 99 (10.5), 98 (13.5), 85 (13.5), 72 (4.8), 55 (2.6).

*Epinorgalanthamine* (**6**). The ^1^H- and ^13^C-NMR spectra data were as reported by Bastida *et al*. [32].

### 3.5. X-ray Structure Analysis Summary

Crystal data: C_17_H_21_NO_3_ from acetone, crystal dimensions: 0.4 × 0.16 × 0.6 mm prism colorless, crystal system: monoclinic P2_1_, cell parameters *a* = 5.8384, *b* = 15.755(2), *c* = 8.2887(11) Å, α = 90°, β = 103.161(3)°, γ = 90°, Z = 2, V = 747.39(17) Å^3^, D_calc_ = 1.285 Mg/m^3^, μ = 0.088 mm^−1^, F(000) = 308, Data collection: Bruker Smart APEX AXS CCD area detector/omega scan, index ranges −18 ≤ h ≤ 6, −18 ≤ 16, −9 ≤ 1 ≤ 9, 6039 collected and 1346 observed reflections. Solution and refinement: The structure was solved by direct methods and refined by full-matrix least squares. Final values are: R1 = 0.316, WR_2_ = 0.0690. Largest diff. peak and hole: 0.140 and −0.120 eÅ^−3^, GooF = 0.969. Supplementary data for compound **1** has been deposited at the Cambridge Crystallographic Data Centre. Copies of this information are available free of charge on request from The Director, CCDC, 12 Union Road, Cambridge CB2 1EZ, UK (fax: +44 1223 336 033, email: deposit@ccdc.ac.uk or internet address http://www.ccd.cam.ac.uk) quoting the deposition number CCDC 678548.

### 3.6. Molecular Modelling

Modelling was performed in gas phase with help of the HyperChem 6.02 version of the MM+ program (University of Moncton). The computational details are presented in reference [33].

### 3.7. Acetylcholinesterase Inhibition Assay

The assay for measuring acetylcholinesterase (AChE) inhibitory activity was performed in 96 well microtitre plates as previously described [14,15]. EC_50_ values are means ± SD of three determinations performed with 10^−8^, 10^−7^, 10^−6^, 10^−5^, 10^−4^, and 10^−3^ M for each tested compound. Acetylcholinesterase from *Electrophorus electricus* L. (electric eel) type V-S lyophilized powder, acetyl-thiocholine iodide (ATCI), and 5,5′-dithio-bis[2-nitrobenzoic acid] (DNTB) were obtained from Sigma Aldrich Chemie (Stenheim, Germany). Fifty microliters of AChE solution (500 U/mL) in phosphate buffer (8 mM K_2_HPO_4_, 2.3 mM NaH_2_PO_4_, 0.15 M NaCl, 0.05% Tween 20, pH 7.6), and 50 μL of the compound dissolved in the same buffer were added to each of the wells. The plates were incubated for 30 min at room temperature before addition of 100 μL of the substrate solution (0.1 M NaH_2_PO_4_, 0.5 M DNTB, 0.6 mM ATCI in Millipore water, pH 7.5). Absorbance was recorded in a Labsystems Microplate Reader (Helsinki, Finland) at 405 nm after 6 min. Enzyme activity was calculated as percentage compared to an assay using a buffer without the tested compound. The AChE inhibitory data were analysed with the Prism software package (Graph Pad Inc., San Diego, USA).

### 3.8. Antiviral and Cytotoxicity Assays

Anti-HIV activity and cytotoxicity were evaluated in human MT-4 cells based on viability of cells that have been infected or not infected with HIV-1 following exposure to various concentrations of the test compound according to the procedure previously described [35,36]. After proliferation for 5 days the number of viable MT-4 cells was quantified by tetrazolium-based colorimetric method (MTT). The HIV-1 NL4-3 virus used was a molecular clone obtained from the National Institute of Health (Bethesda, USA) that was propagated and tittered in MT-4 cells. All anti-HIV evaluations were done in triplicate in two separate experiments.

## 4. Conclusions

The bulbs and aerial parts of *Zephyranthes concolor* contain the alkaloids chlidanthine, galanthamine, galanthamine N-oxide, lycorine, galwesine, and epinorgalanthamine. The structure of chlidanthine was determined by 1D and 2D high resolution ^1^H-, ^13^C-NMR spectra, and X-ray diffraction. Minimum energy conformation for structures of galanthamine, and its structural isomer chlidanthine were calculated by molecular modelling, suggesting structure solvatation plays a role in the conformation distributions. Chlidanthine and galanthamine N-oxide inhibited electric eel acetylcholinesterase, but were about five times less potent than galanthamine, while galwesine was inactive at 10^−3^ M. Inhibitory activity of HIV-1 replication, and cytotoxicity of the isolated alkaloids were evaluated in human MT-4 cells; however, the alkaloids showed poor activity as compared with standard anti-HIV drugs, although most of them were not cytotoxic.

## Figures and Tables

**Table 1:** ^1^H- and ^13^C-NMR spectral data of chlidanthine (**1**), δ in ppm, *J* in Hz.

Position	^1^H * (*J*, Hz)	NOESY	^13^C **	HMBC ***
1	4.50 *(td*, *J* = 3.3, 1.2)	H(2*α*), H(2*β*), H(11*α*)	87.2 *s*	C(11)
2α	1.99 (*ddd*, *J* = −15.9, 5.8, 3.3)	H(1), H(3)	28.1 *t*	
2β	2.59 (*ddd*, *J* = −15.9, 3.3, 1.5)	H(1)		
3	3.79 (*m*, *J* = 5.8, 1.5)	H(2α)	70.0 *s*	C(4), C(1), OMe
4	5.98 (*ddd*, *J* = 10.4, 4.5, 1.2)	H(4a)	124.3 *s*	C(2), C(10b)
4a	6.17 (*dt*, *J* = 10.4, 1.2)	H(4), H(6β), H(12β)	129.4 *s*	C(1), C(3), C(10b)
6α	3.62 (*d*, *J =* −15.0)	H(7), H(12*α),* NMe	60.6 *t*	C(10a), C(6a), C(7), C(12), NMe
6β	4.09 (*d*, *J* = −15.0)	H(4a), H(12*β*)		C(10a), C(6a), C(7), C(12), NMe
6a			128.7 *s*	
7	6.47 (*d*, *J =* 8.0)	H(6*α*), H(8)	121.9 *d*	C(10), C(10ª), C(6)
8	6.60 (*d*, 8.0)	H(7)	115.3 *d*	C(9), C(10), C(6a)
9			145.4 *s*	
10			140.1 *s*	
10a			132.1 *s*	
10b			48.6 *s*	
11α	2.10 (*dt*, *J =* −13.3, 3.0)	H(1)	34.6 *t*	C(10b)
11β	1.52 (*ddd*, *J* = −13.3, 3.8, 1.6)	H(12*α*)		
12α	3.03 (*d*, *J =* −14.3)	H(6α), H(11*β*), NMe	54.0 *t*	
12β	3.28 (*d*, *J =* −14.3, 1.4)	H(4a), H(6*β*)		
OMe	3.37 *s*		56.8 *q*	C(3)
NMe	2.36 *s*	H(6*α*), H(11*α*), H(12*α*)	41.9 *q*	C(6), C(12)

* 500 MHz/CDCl_3_; ** 125 MHz/CDCl_3_; *** ^3^J & ^4^J.

**Table 2:**
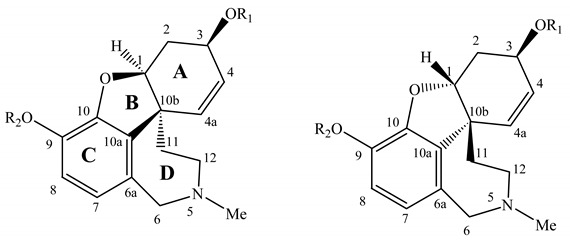
Minimum energy conformation calculated by molecular modelling of chlidanthine (**1**), galanthamine (**2**) and hypothetical structures **1a**, **2a**, **7**, **7a**, **8**, **8a**.

	Config.	Structure	kcal/mol	Config.	Structure	kcal/mol
	**1*S*, 10b*S***			**1*R*, 10b*R***		
**R_1_: Me**	*N*-Meα	**1**	40.57	*N*-Meα	**7**	44.62
**R_2_: H**	*N*-Meβ	**1a**	39.58	*N*-Meβ	**7a**	44.25
**R_1_: H**	*N*-Meα	**2**	38.51	*N*-Meα	**8**	44.08
**R_2_: Me**	*N*-Meβ	**2a**	39.49	*N*-Meβ	**8a**	43.69

**Table 3:** Acetylcholinesterase inhibitory activity of *Z. concolor* alkaloids.

Alkaloid	EC_50_ (M)
Chlidanthine (**1**)	2.41 ± 0.50 × 10^−5^
Galanthamine (**2**)	5.16 ± 1.08 × 10^−6^
Galanthamine *N*-oxide (**3**)	2.62 ± 0.55 × 10^−5^
Galwesine (**5**) > 10^−3^	

EC_50_ values are means ± SD of three determinations (10^−8^ to10^−3^ M).

**Table 4:** Antiviral activity (HIV-1) of *Z. concolor* alkaloids (μg/mL).

Compound	EC_50_^a^ (μg/mL)	CC_50_^b^ (μg/mL)
	HIV-1 (NL4-3)	No virus
Chlidanthine (**1**)	>25	>25
Galanthamine (**2**)	>25	>25
Galanthamine *N*-oxide (**3**)	>25	>25
Lycorine (**4**)	>0.5	0.48
Galwesine (**5**)	>71.4	71.4
Epinorgalanthamine (**6**)	>13	13
AZT **^c^**	0.0007	>1
AMD3100 ^c^	0.001	>5
Nevirapine ^c^	0.034	>2

^a^ EC_50_: Effective concentration needed to inhibit 50% HIV-induced cell death; ^b^ CC_50_: Concentration needed to induce 50% death of non-infected cells; ^c^ Positive control.

## Supplementary Materials

Supplementary materials can be accessed on: http://www.mdpi.com/1420-3049/16/11/9520/s1.

## References

[1] Fernández-Alonso J.L., Groenendijk J.P. (2004). A new species of *Zephyranthes* Herb. S. L. (Amarylli-daceae Hipperastreae), with notes on the Genus in Colombia. Rev. Acad. Colomb. Cienc..

[2] Cano-Santana Z., Castillo-Arguero S., Martinez-Orea Y., Juarez-Orozco S. (2008). Análisis de la Riqueza Vegetal y el Valor de Conservación de Tres Áreas Incorporadas a la Reserva Ecológica del Pedregal de San Ángel, Distrito Federal (México). Bol. Soc. Bot. Méx..

[3] Boit H.G., Döpke W., Stender W. (1958). Alkaloide aus *Hippeastrum rutilum, Zephyranthes andersoniana*, und *Sternbergia fischeriana*. Naturwissenschaften.

[4] Boit H.G., Ehmke H. (1955). Amaryllidaceae alkaloids. VIII. Alkaloids from *Sprekelia formosissima, Galanthus elwesii*, *Zephyranthes candida*, and *Crinum powellii*. Chem. Ber..

[5] Pettit G.R., Cragg G.M., Singh S.B., Duke J.A., Doubek D.L. (1990). Antineoplastic Agents, 162. *Zephyranthes candida*. J. Nat. Prod..

[6] Kojima K., Mutsuga M., Inoue M., Ogihara Y. (1998). Two alkaloids from *Zephyranthes carinata*. Phytochemistry.

[7] Herrera M.R., Machocho A.K., Brun R., Viladomat F., Codina C., Bastida J. (2001). Crinane and Lycorane Type Alkaloids from *Zephyranthes citrina*. Planta Med..

[8] Ghosal S., Shibnath S.K., Unnikrishnan S.G. (1987). Chemical Constituents of Amaryllidaceae. Part 25. Phosphatidylpyrrolophenanthridine Alkaloids from *Zephyranthes flava*. Phytochemistry.

[9] Pettit G.R., Gaddamidi V., Cragg G.M. (1984). Antineoplastic Agents, 105. *Zephyranthes grandiflora*. J. Nat. Prod..

[10] Rao R.V.K., Rao J.V., Seshagiri L.N. (1979). Occurrence of the Rare Alkaloid Maritidine *in Zephyranthes robusta* and *Z. sulphurea*. Curr. Sci..

[11] Ghosal S., Razdan A., Razdan S. (1985). Chemical constituents of Amaryllidaceae. Part 9. (+)-Epimariti-dine, an alkaloid from *Zephyranthes rosea*. Phytochemistry.

[12] Döpke W. (1965). Constitution and Configuration of Tubispacine, an Amaryllidaceae Alkaloid. Archiv der Pharmazie und Berichte der Deutschen Pharmazeutischen Gesellschaft.

[13] Bartolucci C., Perola E., Pilger C., Fels G., Lamba D. (2001). Three-dimensional Structure of a Complex of Galanthamine (Nivalin®) with Acetylcholinesterase from *Torpedo californica*: Implications for the Design of new Anti-Alzheimer Drugs. Proteins.

[14] Berkov S., Codina C., Viladomat F., Bastida J. (2008). *N*-Alkylated galanthamine Derivatives: Potent Acetylcholinesterase Inhibitors from *Leucojum aestivum*. Bioorg. Med. Chem. Lett..

[15] López S., Bastida J., Viladomat F., Codina C. (2002). Acetylcholinesterase Inhibitory Activity of some Amaryllidaceae Alkaloids and *Narcissus* Extracts. Life Sci..

[16] Szlávik L., Gyuris A., Minárovits J., Forgo P., Molnár J., Hohmann J. (2004). Alkaloids from *Leucojum vernum* and Antiretroviral Activity of Amaryllidaceae Alkaloids. Planta Med..

[17] Lin L.Z., Hu S.F., Chai H.B., Pengsuparp T., Pezzuto J.M., Cordell G.A., Ruangrungsi N. (1995). Lyco-rine Alkaloids from *Hymenocallis littoralis*. Phytochemistry.

[18] Ghosal S., Shanthy A., Kumar A., Kumar Y. (1985). Palmilycorine and lycoriside: Acyloxy and acyl-glucosyloxy alkaloids from *Crinum asiaticum*. Phytochemistry.

[19] Min B.S., Kim Y.H., Tomiyama M., Nakamura N., Miyashiro H., Otake T., Hattori M. (2001). Inhibitory effects of Korean plants on HIV-1 activities. Phytother. Res..

[20] Boit H.G. (1956). Alkaloide von *Chlidanthus fragrans*, *Vallota purpurea*, *Nerine undulata* und *Hippeastrum vittatum* (XI. Mitteil. über Amaryllidaceen-Alkaloide). Chem. Ber..

[21] Boit H.G., Döpke W., Stender W. (1958). New Amaryllidaceae Alkaloids. Naturwissenschaften.

[22] Boit H.G., Döpke W. (1960). Alkaloids from *Hippeastrum aulicum* var. *robustum*. Naturwissenschaften.

[23] Boit H.G., Ehmke H. (1957). XIV. Mitteil. über Amaryllidaceen-Alkaloide. Über Chlidanthin, Narcissamin und Nerinin. Chem. Ber..

[24] Bhandarkar J.G., Kirby G.W. (1970). Structure and Biosynthesis of Chlidanthine. J. Chem. Soc. C.

[25] Döpke W., Dalmer H. (1965). Alkaloide aus Amaryllidaceen. Naturwissenschaften.

[26] Peeters O.M., Blaton N.M., De Ranter C.J. (1997). (−)-Galanthaminium Bromide. Acta Crystallogr. C.

[27] Carrol P., Furst G.T., Han S.Y., Joullié M. (1990). Spectroscopic studies of Galanthamine and Galanthamine Methiodide. Bull. Soc. Chim. Fr..

[28] Greenblatt H.M., Kryger G., Lewis T., Silman I., Sussman J.L. (1999). Structure of Acetylcholinesterase Complexed with (−)-Galanthamine at 2.3Å Resolution. FEBS Lett..

[29] Galván R.V., de Rzedowski G.C., Rzedowski J. (2005). Amaryllidaceae. Flora Fanerogámica del Valle de México.

[30] Kobayashi S., Satoh K., Numata A., Shingu T., Kihara M. (1991). Alkaloids N-oxides from *Lycoris sanguínea*. Phytochemistry.

[31] Latvala A., Onur M.A., Gozler T., Linden A., Kivffak B., Hesse M. (1995). Alkaloids of *Galanthus elwesii*. Phytochemistry.

[32] Bastida J., Viladomat F., Bergoñón S., Fernández J.M., Codina C., Rubiralta M., Quirion J.C. (1993). Alkaloids from *Narcissus leonensis*. Phytochemistry.

[33] Jankowski C.K., Foucher S., Fermandjian S., Maroun R.G. (2005). Study of Peptide Oligomer Derived from HIV-1 Integrase. J. Mol Struct. Theochem..

[34] Singh I.P., Bodiwala H.S. (2010). Recent advances in anti-HIV natural products. Nat. Prod. Rep..

[35] Armand-Ugón M., Clotet-Codina I., Tintori C., Manetti F., Clotet B., Botta M., Esté J.A. (2005). The Anti-HIV Activity of ADS-J1 Targets the HIV-1 gp120. Virology.

[36] López S., Armand-Úgon M., Bastida J., Viladomat F., Esté J.A., Stewart D., Codina C. (2003). Anti Human Immunodeficiency Virus Type 1 (HIV-1) Activity of Lectins from *Narcissus* Species. Planta Med..

