# Supplementary material for: Acetylcholinesterase-inhibiting Alkaloids from Zephyranthes concolor
Source: Molecules. 2011 Nov 15;16(11):9520–33. doi: 10.3390/molecules16119520 (PMC6264317; doi:10.3390/molecules16119520)
Supplement: Supplementary file 1 [file molecules-16-09520-s001.docx]

Synonyms for *Zephyranthes* species chemically studied, as registered by the Missouri Botanical Garden (http://www.tropicos.org/Home.aspx).

*Zephyranthes andersoniana*. Name not found, probably written incorrectly instead of *Zephyranthes andersonii* (Herb. ex Lindl.) Baker: *Habranthus andersonii* Herb. ex Lindl.; *Habranthus tubispathus* (L´Hér.) Traub.

*Zephyranthes concolor* (Lindl.) Benth. & Hook. f.: *Habranthus concolor* Lindl.; *Hippeastrum concolor (Lindl.)* Baker.

*Zephyranthes candida* (Lind.) Herb.: *Amaryllis candida* (Stapf) Traub & Uphof*.*;  *Amaryllis candida* Lindl.; *Amaryllis nivea* Schult. & Schult.f.; *Argyropsis candida* (Lindl.) M. Roem.; *Atamosco candida* (Lindl.) Sasaki; *Atamosco candida* (Lindl.) Small.; *Plectronema candida* (Lindl.) Raf.

*Zephyranthes carinata* Herb.: *Amaryllis carinata* (Herb.) Spreng.; *Atamosco carinata* (Herb.) P. Wilson; *Atamosco carinata* Standl.; *Pogonema carinata* (Herb.) Raf.; *Zephyranthes grandiflora* Lindl.

*Zephyranthes citrina* Baker: *Atamasco eggersiana* (Urb.) Britton; *Zephyranthes eggresiana* Urb.

*Zephyranthes flava* (Herb.) Baker: *Pyrolirion flavum* Herb.; *Zephyranthes beustii* Schinz.

*Zephyranthes grandiflora* Lindl.: *Zephyranthes carinata* Herb.; *Zephyranthes macrosiphon* Baker.

*Zephyranthes robusta* (Herb. Ex Sweet) Baker.: *Habranthus robustus* Herb. Ex Sweet.

*Zephyranthes rosea* Lindl.: *Amaryllis carnea* Schult.f.; *Amaryllis rosea* (Lindl. ) Spreng.

*Zephyranthes sulphurea* hort. Name not found, probably an horticultural name.

*Zephyranthes tubispatha* (L´Hér.) Herb.: *Amaryllis nervosa* Kunth.; *Amaryllis tubispatha* Ker Gawl.; *Zephyranthes lindleyana* Herb.; *Zephyranthes mesochloa* Herb.; *Zephyranthes nervosa* (Kunth) Herb.
